# Mechanical Damage Caused by Compression and Its Effects on Storage Quality of Mandarin

**DOI:** 10.3390/foods13060892

**Published:** 2024-03-15

**Authors:** Haoyu Tian, Hong Chen, Xiaoxian Li

**Affiliations:** 1 College of Engineering, Huazhong Agricultural University, Wuhan 430070, China; tian_haoyu@webmail.hzau.edu.cn (H.T.); xiaoxian1@webmail.hzau.edu.cn (X.L.); 2 Key Laboratory of Agricultural Equipment in Mid-Lower Yangtze River, Ministry of Agriculture, Wuhan 430070, China

**Keywords:** compression, mechanical damage, image segmentation, storage quality, regression model

## Abstract

Mandarin is vulnerable to a range of external loads during processing and shipping, which can cause interior mechanical damage that can happen right away or over time and cause serious rotting when kept in storage. In this study, mandarin was treated to a certain quantity of compression load that did not result in a noticeable rupture of the peel. The interior pulp structure of mandarin was examined for damage prior to peel damage using CT scanning and image reconstruction. An image segmentation method based on mask processing was then used to calculate the pulp damage rate. We examined the variations in physiological activities and internal components between the test group that underwent compression load and the control group that did not undergo this type of stress during storage. The aim was to investigate the factors that contributed to the faster decay of mandarin following mechanical damage. Regression analysis was also used to establish a quantifiable relationship between the amount of compression deformation and the rates of damage and decay of mandarin during storage. The findings demonstrated that mandarin pulp exhibited visible mechanical damage when compression deformation exceeded 8 mm. This led to the disruption of physiological processes like respiration and polysaccharide breakdown, which in turn decreased the hardness of the fruit and sped up its rotting. This study identifies the critical range of compression deformation that leads to the beginning of pulp damage in mandarins. Additionally, it clarifies the quality deterioration mechanism of mandarins that have been subjected to compression damage during the storage period. Therefore, in practical production, various methods of picking, sorting, and collecting mandarins can be optimized to control the amount of compression deformation within a suitable range. This will reduce the probability of pulp damage. According to the study’s conclusions, storage conditions can be optimized to regulate the physiological activities of mandarins in a targeted manner. This can minimize the probability of fruit decay and reduce economic losses.

## 1. Introduction

Mandarins such as Satsuma mandarin and Ponkan have loose, thin, and easily removed peel, and they are important cultivated varieties in China [1]. Before being sold to customers, fresh mandarin typically goes through harvesting, transportation, and storage, all of which expose the fruit to a range of external loads. Mandarin may be subjected to excessive clamping forces during manual or robotic picking. Additionally, stacked placement of fruit during collection and sorting can create compression loads. Studies have shown that external forces can easily cause sudden or slow changes in the local texture of the fruit, thus resulting in immediate or delayed mechanical damage [2]. Mandarin pulp may be more impacted after being compressed because the peels are elastic and the pulp is moist and tender. At the same time, the mandarin epidermis contains a soft secretory vesicle called the oil gland. This structure is susceptible to rupture when exposed to external forces, leading to mechanical damage and an increased risk of pathogen invasion. As a result, the quality of the internal pulp is threatened [3]. Even if the pulp is damaged, this type of citrus damage is visually indistinguishable from healthy citrus due to the presence of the exocarp. This type of citrus may have a faster decay rate during subsequent storage, resulting in complete damage before it is marketed, which can cause serious losses [4]. According to reports, the annual fruit loss rate in post-harvest distribution in China ranges from 15% to 25% and can sometimes be as high as 40%. This is attributed to insufficient research on post-harvest technical problems of fruits and vegetables [5]. Identifying the potential sources and primary characteristics of mechanical damage is the initial step in optimizing post-harvest fruit protection techniques.

Currently, research on mechanical damage of fruits and vegetables is a hot topic. The damage sources in the actual situation are simulated by different mechanical test methods such as compression and shear, and the corresponding mechanical characteristic parameters or damage limit parameters are calculated according to the load-displacement curve to evaluate the anti-damage performance of different fruits. Lin et al. investigated changes in the mechanical parameters, such as the breaking limit of reticulated mandarins with storage time by performing periodic squeeze tests on reticulated mandarins during storage periods [6]. Lu et al. investigated the mechanical damage limit of rose balsam oranges by TPA and puncture tests and found that their equatorial parts were relatively weak in terms of compressive capacity [7]. To forecast fruit damage, a number of studies have looked into the relationship between external load and fruit damage volume. For example, in their compression experiments, Razavi et al. investigated the link between damage volume and the loading of pear fruits under various force loads [8]. Piotr Komarnicki et al. investigated the changes in surface pressure values and bruising of ‘Golden Crown’ apples when impacted by different rigid plates at varying drop heights [9]. However, mechanical damage in mandarin is relatively complex, and damage to either the peel, flesh, or whole fruit can lead to severe decay in subsequent storage. In order to establish a relationship between external stresses and the degree of damage to mandarin, it is first necessary to clearly define the different damage characteristics of mandarin. The percentage of significantly damaged mandarin in a drop test has been defined by some scholars as the rate of mechanical damage to the entire batch of mandarin [10]. It has also been found that when mandarin is mechanically damaged, the peel produces an essential oil that contains polymethoxy flavonoids. This can be detected by fluorescence imaging, and the damaged area of the peel can be determined [11]. However, few of the available studies have addressed internal pulp damage in mandarin. This will be an important direction for future research.

On the other hand, changes in physiological activity and internal compositional content of the fruit after being subjected to external loads directly determine whether the fruit can continue to be processed or marketed. Therefore, some studies have been conducted to investigate the changes of internal and external components of fruits under external load influences. For example, Santos et al. examined the effects of damages caused by fresh-cut, compression, and impact on the quality of Rosa oranges during storage and found that damage had significant effects on the weight loss rate, soluble solid content, titratable acid content, and pH of oranges [12]. Miranda et al. evaluated the effect of impact damage on the quality of citrus after harvest and found that the impact damage of oranges resulted in lower soluble solids and titratable acidity than the control group [13].

In the existing damage identification research, some scholars have studied the microscopic image characteristics of mandarins after blanching, and the change rules of microstructure, such as stomata of mandarin peel, with the increase in blanching time was clarified [14]. Some scholars have proposed a method for detecting the mechanical damage of sweet lemon by using image processing technology and ultraviolet radiation. The image is captured under ultraviolet light with a wavelength of 6nm, and the number of oily spots on the peel is defined as the degree of mechanical damage [15]. Some scholars extracted the image boundaries of citrus damage patterns using an improved algorithm, merged and labeled them, and generated an image of the external damage patterns of citrus [16]. Doosti-Irani et al. demonstrated that a thermal imaging system can distinguish the relationship between internal and external temperatures of damaged fruit [17]. The above research shows that it is feasible to analyze and evaluate the mechanical damage of fruits by using image features. On the other hand, significant mechanical injury to the mandarin fruit’s peel or pulp speeds up the deterioration of mandarin quality. It is still difficult to identify the correlation between pulp damage and the decline in mandarin quality during storage due to the variety of pulp damage types and the complexity of damage features. It remains to be investigated to what extent compression force or deformation can maintain mandarin quality in storage within a manageable range.

Therefore, this study used CT scanning and image processing techniques to detect the damage sequence and characteristics of mandarin peel and pulp, and then measured the degree of pulp damage in order to investigate the damage changes of mandarin peel and pulp under the effect of different compression deformation. We observed quality and physiological activity changes in both healthy and squeezed mandarin throughout storage in order to shed light on the connection between squeezing damage and mandarin quality degradation. Regression analysis was used to establish quantifiable connections between compression deformation and the rates of pulp damage and storage decay in order to determine the range of compression deformation that would guarantee citrus quality would stay stable during storage. Our findings will provide a theoretical basis for reducing the storage decay rate of mandarin.

## 2. Materials and Methods

### 2.1. Materials and Pretreatment

Satsuma mandarin used in this experiment was harvested from Ninggang Mandarin General Farm, Jiangxia District, Wuhan City, Hubei Province, China (29°58′ N–31°22′ N, 113°41′ E–115°05′ E). The individual responsible for the planting base evaluated the appearance and taste of the mandarin and measured some quality indicators. Based on this evaluation, a batch of ripe mandarins suitable for picking and storage was selected. The following are the specific criteria: Ripe mandarin have an orange-yellow epidermis and almost no green on the surface. They emit a distinct flavor caused by trace volatile substances and have a pleasant taste without bitterness. At the same time, a portion of the mandarin was tested to determine the ratio of soluble solids to titratable acid (Section 2.6 describes the instruments and methods used for testing). A ratio of 8:1 or higher indicates that the fruit is ripe for picking and storage. We then chose samples with a pleasing appearance and no signs of pests or diseases for testing. In order to minimize the influence of individual differences, mandarin with transverse diameter of 60~70 mm, height of 50~55 mm, consistent size, and uniform shape were selected.

150 healthy mandarins were selected for image feature study (divided into a control group and four treatment groups, 30 mandarins in each group), and 225 healthy mandarins were selected for storage experiment (divided into a control group and four treatment groups, 45 mandarins in each group).

In order to compare the image features of mandarin pulp and peel before and after compression, the CT scan images and 50-times-enlarged images of the mandarins of the four treatment groups in the image group were collected first. The specific image acquisition methods are described later.

### 2.2. Damage Sample Preparation

**Figure 1:**
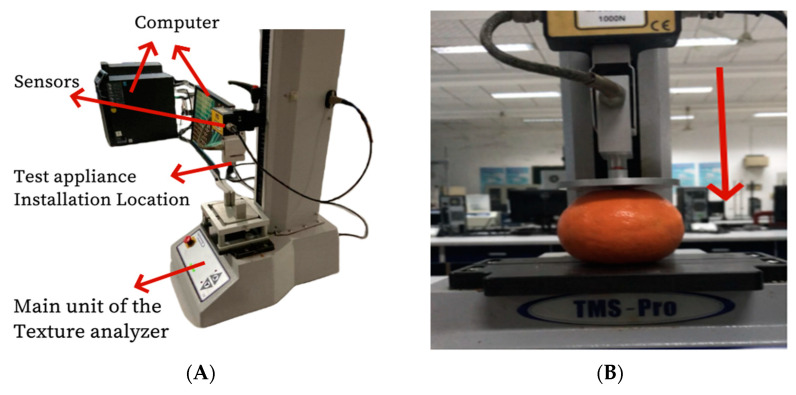
Compression equipment and methods. (**A**) Test equipment; (**B**) Compression method. Note: Red arrows indicate the direction of compression.

Studies that have already been conducted indicate that when the compression deformation was approximately 20 mm, over 95% of mandarin peels demonstrated considerable rupture [18]. With powerful functions, high detection accuracy, and stable performance, the Texture Analyzer is a powerful analytical tool for studying the physical properties of foods. Using the TMS-Pro Texture Analyzer (Food Technology Corporation (FTC), Sterling, VA, USA, Figure 1A), four treatment groups were exposed to varying degrees of compression deformation (4 mm, 8 mm, 12 mm, and 16 mm) in order to examine the changes in peel and pulp damage prior to rupture and to guarantee the storability of mandarin. The ambient temperature in the test laboratory was 22 °C, and the relative humidity was 58% (by Hengge HK-J8A102/HK-J8A103 Multi-function Digital Temperature and Humidity Meter, Shenzhen, China). The instrument parameters were set as follows: rate before test 30 mm/min, rate during test 60 mm/min, return rate after test 300 mm/min, trigger force 0.1 N. After extruding downward from the peduncle area and achieving the appropriate amount of deformation, the texture analyzer was allowed to stand for 10 s (Figure 1B).

### 2.3. Image Acquisition of Mandarin Internal Structure

CT scanning technology is able to scan and reconstruct the internal structure of the fruit, which is useful for studying internal damage in mandarin. In order to observe the changes in the pulp after compression, 120 mandarin fruits from 4 treatment groups were scanned by micro CT nano Voxel-2000 CT machine (Sanying Precision Instruments Co., Ltd., Tianjin, China),with CT scanning parameters of pixel matrix of 1920 × 1536 and flat panel detector pixel size of 0.1 μm. Mandarin samples were placed on the scanning platform for automatic 360° rotation scanning, and 1080 images (in 32-bit Tiff format) were obtained after each scan. The Voxel Studio V2.5.1.25 software was used to reconstruct the original file obtained by CT scan. The reconstructed structure views of the cross-section and the longitudinal section were selected. Repeated observation was conducted on 30 samples at each level of compression deformation. The images under different compression deformations were analyzed into four typical damage categories.

### 2.4. Microscopic Image Acquisition of Exocarp

In order to observe the compressed surface of the samples more clearly and further clarify the changes in the mandarin epidermis after compression, a high-resolution digital microscope (VHX-6000, Keyence (China) Co., Shanghai, China) was used to collect the enlarged images of the epidermis of all treatment groups after compression with a magnification of 50 times. The images of the epidermis surrounding the fruit stem were collected. During the collection process, there was no natural light condition in the room. The light source was provided by the microscope, and the same light condition was ensured. Repeated observation was conducted on 30 samples at each level of compression deformation. The images under different compression deformations were analyzed into four typical damage categories.

### 2.5. Electron Microscope Image Acquisition of Exocarp

In order to explore the changes of the microstructure of mandarin peel before and after compression, the structure of the stomata of mandarin peel in the control group and the experimental group was observed. The outer epidermis of mandarin samples was cut with a thin blade to avoid scratching the observation surface. The peel samples were refrigerated in a fixative solution (0~4 °C) and made to stand for 24 h. The samples were dehydrated with 30%, 50%, and 70% ethanol solutions for 15 min and then dehydrated with 90% and 100% ethanol solutions for 10 min. The samples were soaked in 100% isoamyl acetate solution for 20 min. Subsequently, the samples were air-dried in a PP fume hood in a test chamber with an ambient temperature of 20 °C and a relative humidity of 58%. After the samples were dried, they were placed in a JFC-160 ion sputtering apparatus (Tokyo Electron Co., Ltd., Tokyo, Japan) for gold plating. After gold plating, the mandarin peel samples were observed under JSM-6390/LV scanning electron microscope (Tokyo Electron Co., Ltd., Tokyo, Japan). Because the electron microscope observation needs to take part of the peel for sample preparation, the microscopic images of the peel after compression are compared and analyzed with the microscopic images of the control group. Repeated observation was conducted on 30 samples at each level of compression deformation. The images under different compression deformations were analyzed into four typical damage categories.

### 2.6. Storage Test

Mandarin should be stored for one to two months after harvest before being sold. On the other hand, later phases of storage may see an acceleration of mandarin deterioration due to mechanical damage from compression. This study simulated actual mandarin storage conditions by packing each mandarin in PE film bags with good air and water permeability (0.01 mm thickness) and storing the mandarin for 40 days at ambient temperature (20~25 °C) in a dark environment to examine quality changes of compressed mandarin during storage. Changes in the amount of decomposed mandarin, respiration intensity, soluble solids (TSS) content, titratable acid (TA) content, and hardness were all observed throughout this storage time. On the day of testing, these indexes were measured once and then every ten days. The measurement method of each indicator was as follows:

Firmness: According to Mazidi et al. [19], a texture analyzer (TMS-Pro, Food Technology Corporation (FTC), Sterling, VA, USA) was used to measure the firmness of fruits. After peeling the fruit samples, an 8 mm diameter cylindrical probe was used for the hardness test, and its set rates were 30 mm/min prior to the test, 25 mm/min throughout the test, and 9 mm for the puncture depth. Five mandarins were randomly selected from each group to be measured, and the average value was used as the hardness of the mandarin in that group. The first peak force as the hardness of the mandarin was taken.

Respiration rate: According to Li et al. [20], four fruits were randomly selected from each group and placed into a sealed plastic box for 3 h. Then, 1.0 mL of gas inside was extracted with a gas-tight needle. A gas chromatograph (7890A, Agilent Technologies Inc., Santa Clara, CA, USA) was used to determine the content of the released CO_2_. The respiration rate was calculated by the following formula:

$$\mathrm{Respiration}\;\mathrm{rate}\;\left(\mathrm{mL}\cdot {\mathrm{kg}}^{-1}\cdot h^{-1}\right)=\frac{(C_{1}-C_{2})\times V}{m\times t}$$

Note: C_1_ is the concentration of CO_2_ in the sealed box the sample is in, C_2_ is the concentration of CO_2_ in the empty box, V is the volume of the container, m is the mass of the fruit, and t is the time of sealing.

Total soluble solid (TSS) content: Five fruits randomly selected from each group were compressed. After compression, TSS content was determined with a portable hand-held digital refractometer (PAL-1, ATAGO Co., Kobe, Japan). Each group was measured three times, and the average was calculated.

Titratable acid (TA) content: According to AOAC standard [21], 5 fruits randomly selected from each group were compressed. After compression, TA was determined in percentage of citric acid by titrating NaOH 0.1 N into 10 mL of juice diluted in 90 mL distilled water until pH 8.1.

Decay: Additional 20 fruits from per group were used to measure the amount of decay. Decay was determined in terms of the number of fruits with visible pathogenic symptoms by visual observation of green or blue *Penicillium* sp. molds.

### 2.7. The Establishment Method of Prediction Model

Since the degree of mechanical damage of mandarin largely affects the decay rate during the subsequent storage period, it is necessary to explore the relationship between mandarin damage characteristics and storage decay rate. As a classical image processing method, image segmentation has a good effect on extracting local features of images and is an important means for further analysis after obtaining images [22]. In this paper, an image segmentation process and method for specific mandarin sectional images was proposed (written in results and analysis). The cavity area in the middle of the pulp before and after compression was extracted, and the difference in pixel area between the two was compared, so as to measure the degree of damage of mandarin. Finally, a quantitative model of the deformation of mandarin in relation to the degree of damage and the rate of storage deterioration was established by linear regression.

### 2.8. Statistical Methods

The experimental data were organized and described statistically using Excel 2013 (Microsoft Corporation, Redmond, WA, USA) software, and statistical analyses such as analysis of variance (ANOVA), LSR multiple comparisons, etc., were performed using SPSS 26.0 software, and graphing was performed using OriginPro 2017 (OriginLab Corporation, Northampton, MA, USA) software.

## 3. Results and Discussion

### 3.1. Image Features of Mandarin Internal Structure

**Figure 2:**
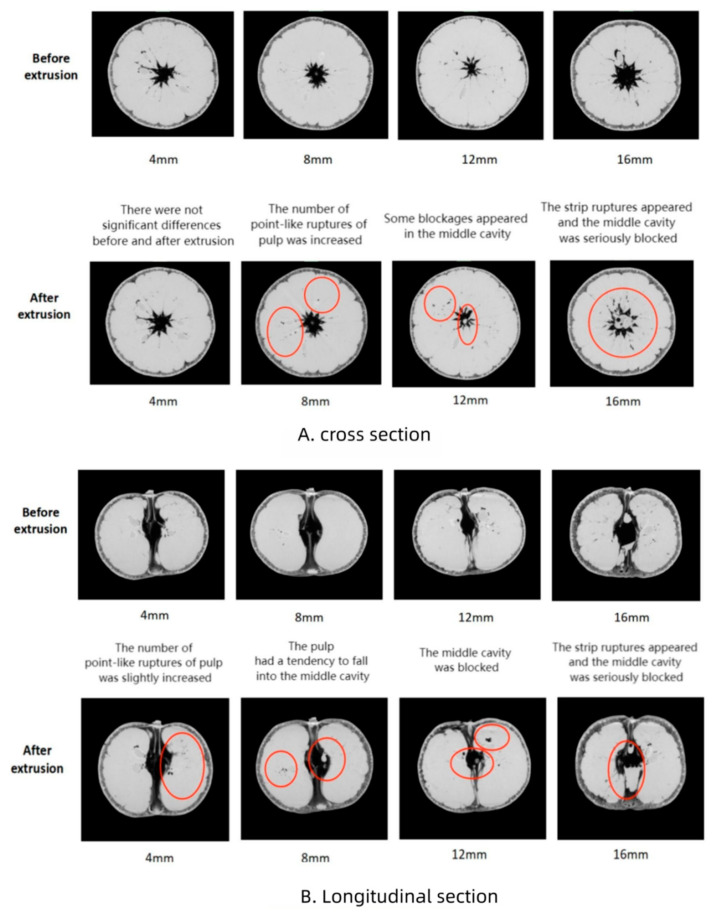
CT scan images of mandarins. The black font in the middle of the picture shows the change of the citrus pulp before and after compression, and the red circle is a mark for highlighting.

The internal structure images of mandarin in different directions are shown in Figure 2A. The analysis showed that when the compression deformation was 4 mm, there was no blockage in the middle cavity of the fruit before and after compression, the cavity in the middle of the pulp increased slightly after compression. There were a few point-like ruptures on the pulp, but there were not significant differences before and after compression. As the amount of compression deformation increases, pulp breakage begins to worsen. When the compression deformation was 8 mm, the number of point-like breaks of pulp was larger after compression than before compression. The longitudinal section (Figure 2B) showed that the pulp had a tendency to fall into the middle cavity. This indicates that the mandarin pulp has gradually begun to damage under this deformation. As the amount of compression deformation continues to increase, the ruptured pulp begins to converge toward the central cavity. New fractures are more likely to occur at existing fractures.

When the compression deformation was 12 mm, the pulp structure was destroyed. The middle cavity increased significantly after compression, and some blockages appeared in the middle cavity. The rupture on the pulp was further increased, and rupture volume began to increase. At this point, if compression is continued, the blockage of the cavity in the center of the pulp and the size of the fracture opening will continue to increase. When the compression deformation was 16 mm, the number of ruptures on the pulp surface increased significantly, some of which changed from point shape to strip shape. After compression, the middle cavity increased more obviously, and there appeared a very obvious blockage; the pulp was severely damaged.

Our study found that compression caused internal damage to mandarin, characterized by pulp structure rupture and central cavity deformation, and the damage is concentrated near the axis of the mandarin. This destruction of pulp occurs prior to the rupture of the pericarp. In this instance, it is more likely to result in the growth of bacteria, which will hasten the breakdown of the juice capsule. This will upset the fruit’s internal environment and physiological processes, which will ultimately hasten the deterioration of quality [23]. Yan et al. discovered that the pulp of mandarin is damaged before the rind when subjected to crush loading, due to its lower ultimate carrying capacity compared to the rind [24].

### 3.2. Magnified Image Characterization of Mandarin Exocarp

**Figure 3:**
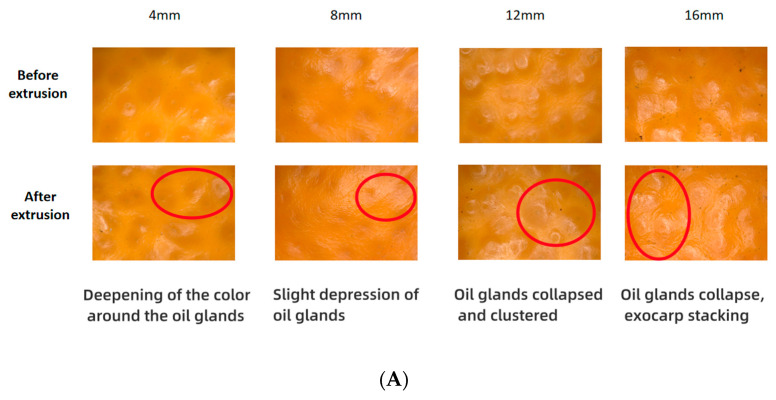

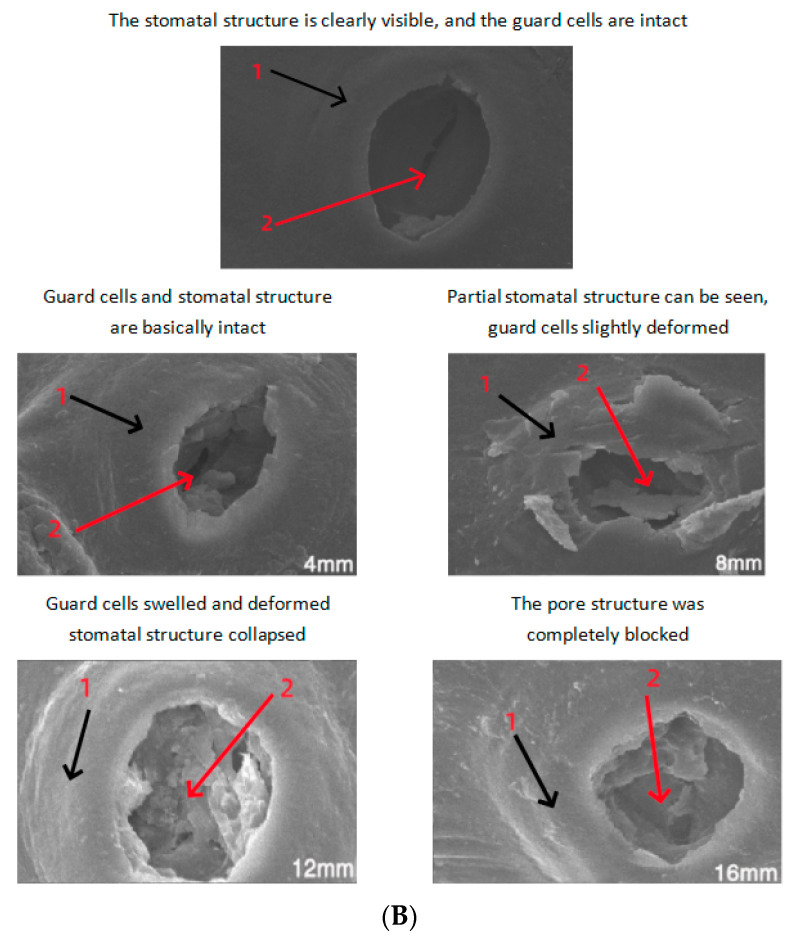
Image acquisition of mandarin peel before and after compression. (**A**) Magnified image characterization of mandarin exocarp; (**B**) Electron microscope image characteristics of exocarp. The corresponding explanation of the markings drawn in the diagram is given at the beginning of Section 3.3 The two crescent cells in the images were a pair of guard cells (labeled as 1), and the cavity enclosed by a pair of guard cells was stomata (labeled as 2).

The enlarged 50 times images of mandarin epidermis are shown in Figure 3A. The surface of mandarin without compression was uniform, smooth, undamaged. Due to the accumulation of essential oil, some oil glands showed a convex surface, and the color difference between oil glands and other parts of the exocarp was small. Other oil glands showed a flat shape, which may have been due to the loss of essential oil during maturation [25]. When the compression deformation was 4 mm and 8 mm, no significant differences in images were observed before and after compression except for slight depression of oil glands on the surface of mandarin after compression. When the compression deformation increased to 12 mm, the whole peel remained flat and uniform, but the degree of depression of the oil glands was deepened after compression, and some oil glands spread and extended towards the surrounding areas under pressure to form an oil gland aggregation region. When the compression deformation increased to 16 mm, obvious wrinkles and folds on the surface of the peel were observed, and the oil glands were extended from the middle to the surrounding, but no obvious epidermal rupture was observed yet.

In summary, within the selected range of compression deformation in this study, there was no compression rupture on the mandarin peel. However, with the increase in compression deformation, the smoothness of the peel was decreased, and the oil glands were gradually depressed under stress. When the compression deformation reached 16 mm, the peel shrank seriously and stacked locally. If the compression continued, the stacked peel was torn apart due to excessive local stress to form damage. It has been found that mechanical damage destroys the integrity of the cell membrane of mandarin peel, causing rupture of the oil cells and inducing rupture of the microstructure of the peel and physiological disorganization [26]. In conjunction with our results, even though the mandarin peel did not show visible breakage under crushing, the epidermal microstructure may have been disrupted, thereby losing its protective effect on the internal structure.

### 3.3. Electron Microscope Image Characteristics of Exocarp

The scanning electron microscope images of mandarin peel are shown in Figure 3B. The two crescent cells in the images were a pair of guard cells (labeled as 1), and the cavity enclosed by a pair of guard cells was stomata (labeled as 2). The overall structure of the stomata on the mandarin peel in the experimental group (4 mm) and the control group was complete and clearly visible, and the guard cells were not damaged. The stomata on the mandarin peel in the 8 mm compression deformation group was slightly damaged but not collapsed, and the location and general structure of the stomata could still be observed. The stomata on the mandarin peels at compression deformation of 12 mm and 16 mm completely collapsed and destroyed, and the guard cells were obviously deformed, and the stomata on the mandarin peels of the latter (16 mm) were more seriously damaged. These results indicated that compression could cause certain damage to the stomatal structure of mandarin epidermis. The more serious the compression, the more serious the damage to the stomatal structure. When the compression deformation was greater than 12 mm, the stomata on mandarin peel was completely destroyed. Scholars have found that mechanical damage accelerates the degradation of mandarin cell walls, which are prone to some degree of deformation before complete degradation, and that this deformation can lead to cell wall collapse or swelling. This is close to our findings [27,28]. The damage to mandarin epidermal tissue will make mandarin more susceptible to bacteria, molds, and other invasions, thus resulting in decreased fruit quality or even decay and shortened fruit shelf life.

### 3.4. Quality of Storage—Firmness

**Figure 4:**
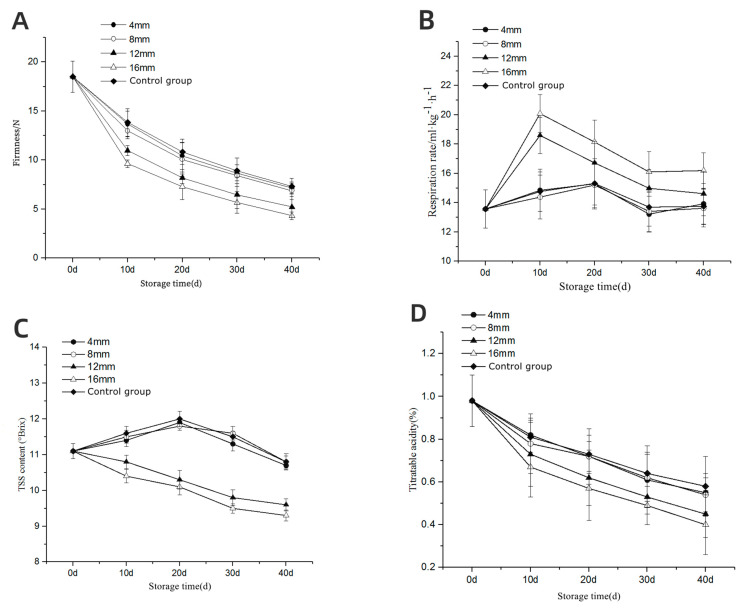

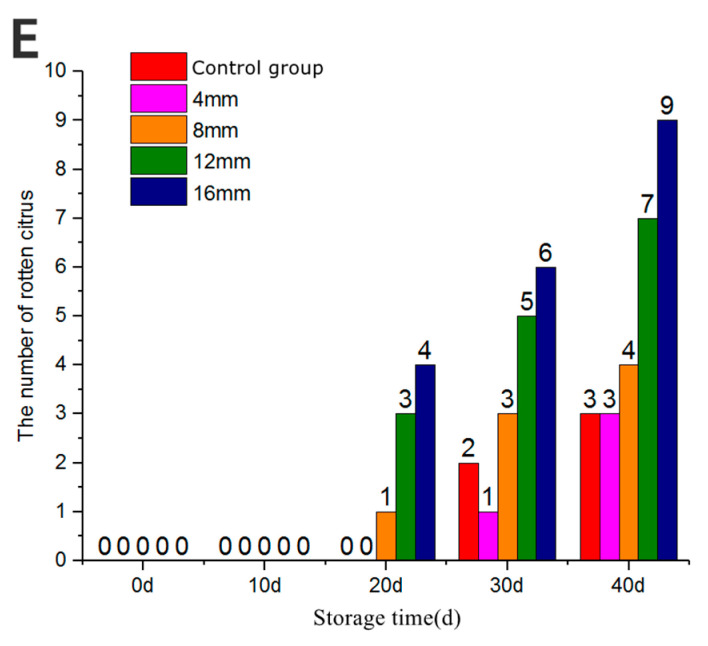
Quality change diagram of mandarin during storage period. (**A**) Variation of firmness with storage time; (**B**) Variation of the respiration rate with storage time; (**C**) Variation of TSS with storage time; (**D**) Variation of TA with storage time; (**E**) Variation of the number of decay with storage time.

As shown in Figure 4A, the firmness of mandarin was gradually decreased with the storage time, and the decline rate gradually slowed down. At day 40 of storage, the firmness of mandarin with deformation of 4 mm, 8 mm, 12 mm, and 16 mm was 2.6%, 6.3%, 28.9%, and 41.0%, respectively, lower than that of the control group. There was no significant difference in the firmness between mandarin fruits subjected to 4 mm or 8 mm compression deformation and those in the control group. The firmness of mandarin with compression deformation of 12 mm and 16 mm was significantly (*p* < 0.05) lower than that of the control group. Our results indicated that mandarin displayed a certain resistance to compression damage, but a compression deformation more than 12 mm would significantly affect the firmness of mandarin, and that the greater the deformation was, the softer the fruits became.

Fruit firmness decreases with extended storage because polysaccharides including pectin, cellulose, and hemicellulose are broken down during fruit ripening [29]. Furthermore, carbohydrases are linked to the breakdown of polysaccharides in fruit cell walls [30]. It has been observed that mechanical injury increases the activities of polygalacturonase and pectin methylesterase, speeding up their breakdown and softening of fruit [31]. This is consistent with our findings and explains why broad-skinned mandarins lose some of their fruit firmness following damage. The cell wall is essential to preserving the cell’s mechanical strength. Mandarin epidermal cells were discovered to be damaged by compression to varied degrees in Section 3.3. This may be one of the primary causes of the loss in firmness of fruits that have compression damage because it decreases the mechanical strength of the fruit’s exocarp.

### 3.5. Respiration Rate

As shown in Figure 4B, during storage, the respiration rate of the mandarin was gradually increased, and then gradually decreased after reaching the peak. At day 40 of storage, the respiration rate of mandarin with compression deformation of 4 mm, 8 mm, and 0 mm was found to be 102.6%, 100.4%, and 101.4%, respectively, as much as that at day 0 with no significant difference (*p* > 0.05). During the first 10 days of storage, the respiration rate of two groups with compression deformation of 12 mm and 16 mm increased significantly. On day 40 of storage, the respiration rate of these two experiment groups was 6.3% and 17.7% higher than that of the control group, respectively, with significant difference (*p* < 0.05). The higher the compression deformation was, the more significant increase in the respiration rate was observed.

It has been reported that vibration and other mechanical damage usually lead to increase in respiration strength in plant tissues, which might be explained by the fact that plant tissues resist damage by strengthening their breathing. Durigan et al. reported a 1.5-fold increase in respiration rate in compression-injured, 2.2-fold in fresh-cut, and 3-fold in impact-injured fruit compared to uninjured fruit. Our results are in general agreement with these findings [32].

### 3.6. Total Soluble Solids (TSS)

As shown in Figure 4C, the soluble solid contents of different groups displayed different trends during storage. TSS contents of mandarin with 4 mm and 8 mm compression deformation and those of the control were increased first, and then were decreased. On day 20 of storage, TSS reached the maximum and then began to decline. On day 40 of storage, the difference in TSS between 4 mm and 8 mm compression deformation groups and the control group was less than 3%. TSS of 12 mm and 16 mm groups showed a decreasing trend. On day 40 of storage, TSS in these two experiment groups was significantly lower than that in the control group (*p* < 0.05).

It was reported that TSS in mandarin fruits was increased during storage, which might be attributed to the fact that the acid in the fruit, as an important substrate in the oxidation process of Krebs cycle, was partially converted into soluble sugar during ripening, causing the increase in TSS during storage [33]. In addition, fruits would lose large amounts of water during storage, resulting in an increase in soluble sugar concentration, which might be another important reason for the increase in TSS [34]. In this study, due to the film bag packaging, the water loss of mandarin in each group was not obvious (less than 5%); thus, the increase in TSS caused by mandarin water loss was negligible. Consequently, the increase in TSS content of 4 mm and 8 mm groups and the control group in the early storage was caused by the conversion of acid in the fruit, rather than by water loss.

TSS contents of 12 mm and 16 mm groups were significantly lower than that of the control group. The main reason might be that the fruit TSS was consumed as energy by fruit respiration after mechanical damage [35]. As shown in Figure 4B, compared with other groups, the respiration rates of 12 mm and 16 mm groups were increased significantly during storage, which resulted in the increased consumption of TSS. Therefore, TSS was decreased at the beginning of storage. Furthermore, TSS of 4 mm and 8 mm groups and of the control group was decreased after 20-day storage. This phenomenon could be explained by the consumption of TSS through respiration.

### 3.7. Titratable Acid (TA) Content

As shown in Figure 4D, the TA content of mandarin in each group gradually decreased with the extension of storage time. On day 40 of storage, TA contents of 4 mm, 8 mm, 12 mm, and 16 mm groups were 5.2%, 6.9%, 22.4%, and 31.0% lower than that of the control group, respectively. The difference in TA content between the 4 mm and 8 mm experiment groups and control group was not significant. TA contents of 12 mm and 16 mm groups were significantly (*p* < 0.05) lower than that of the control group.

TA in the fruit decreased during storage, which is common in fruits such as oranges and lemons [36]. This might mainly be due to the decomposition of the organic acids (mainly citric acid) in the mandarin, part of which were converted into sugar [37].

TA contents of 12 mm and 16 mm groups were observed to be significantly lower than those of other groups. The reason might lie in the fact that when the compression deformation was beyond a certain degree, the irreversible mechanical damage occurred inside the fruit, which destroyed the internal structure, resulting in ascorbic acid (vitamin C), which was exposed to oxygen and caused an oxidation reaction and its degradation [38]. The intensity of oxidation reaction was related to the respiration inside the fruit. As shown in Figure 4B, the respiration rates of 12 mm and 16 mm groups were increased significantly during storage, indicating that the oxidation reaction in the fruit was more intense, and the degradation of ascorbic acid was accelerated. This might explain the significant decrease in TA content. Some scholars have also found consistent conclusions in the study of mechanical damage in peaches [39].

### 3.8. The Number of Decay

The decay of all groups of mandarin during storage is illustrated in Figure 4E. On the 20th day, mandarin in the test group began to exhibit decay, while the control group did not show any signs of decay. As the storage duration increased, the number of decayed samples in the test group also increased. By the 40th day of storage, the total number of decayed fruits in the test group with compression deformations of 12 mm and 16 mm had reached seven and nine, respectively, which was significantly higher compared to the control group. Additionally, the total number of rotten fruits in the test group with 4 mm and 8 mm compression deformations was three and four, respectively, showing no significant difference from that of the control group. Combined with the findings in Section 3.1, Section 3.2, Section 3.3, Section 3.4, Section 3.5, Section 3.6 and Section 3.7, compression deformation greater than 8 mm damaged the cellular structure of the mandarin epidermis and fruit pulp, resulting in physiological disorders such as respiration and ultimately increasing the decay rate.

### 3.9. Prediction Model of Mandarin Decay Rate Based on Image Processing

From the above analysis, it can be seen that for mandarins with different compression deformation, the size of the cavity in the middle of the pulp also changed to varying degrees. Studies have shown that because mandarin is soft and juicy, it is easy to break under load and cause juice outflow. The displacement and deformation of the pulp determine the degree of damage to its internal structure and the degree of quality change [40]. The change in the size of the middle cavity directly reflects the severity of the mandarins being compressed. Therefore, this paper extracts the central cavity of the pulp in the CT scan images by image segmentation methods; the changes of pixel area before and after compression were analyzed, and the flesh damage rate was calculated according to Formula (1).

A = (S2 − S1)/S1 × 100%(1)

Note: A represents the damage rate; S2 represents the area of the middle cavity after compression; S1 represents the area of the middle cavity before compression.

**Figure 5:**
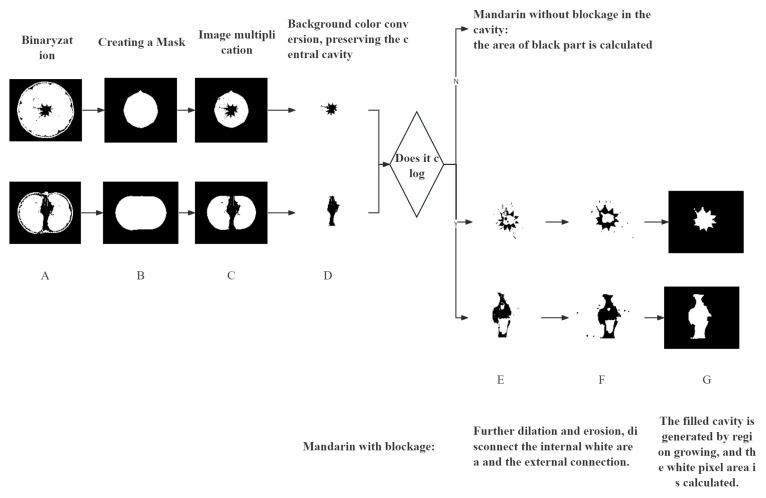
Flow chart of pulp cavity extraction. (**A**) Binaryzation; (**B**) Creating a Mask; (**C**) Image multiplication; (**D**) Background color conversion, preserving the central cavity; (**E**,**F**) Further dilation and erosion, disconnect the internal white area and the external connection; (**G**) The filled cavity is generated by region growing, and the white pixel area is calculated.

In order to accurately extract the cavity in the middle of mandarin pulp, we divided the image into two categories: cavity without blockage and cavity blockage. The image is read and segmented by Matlab-R2018b digital image processing software. The specific process and methods are shown in Figure 5. This method has good applicability to the images of this batch of samples. According to the difference between the cross-sectional and longitudinal cross-sectional images, the mask suitable for different cross-sections can be obtained by changing the size of the erosion core, as shown in Figure 5B. For the cavity without blockage, the black part pixel area can be directly calculated after extraction, which is the cavity area. For the blocked cavity, it is necessary to further select the black and white pixel parts inside the cavity through the region growing algorithm to obtain a fully filled cavity image and calculate the area, as shown in Figure 5G.

**Figure 6:**
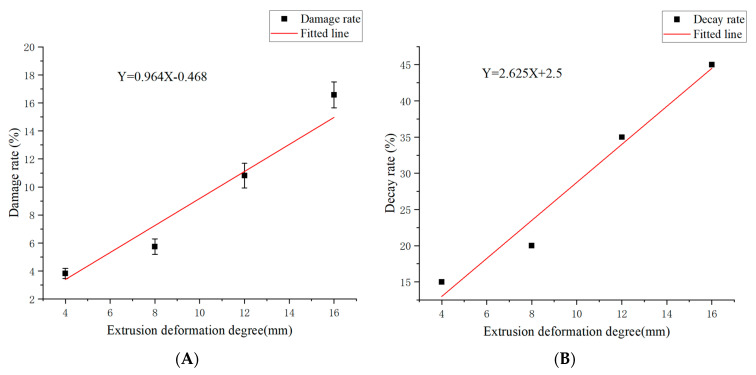
Linear regression of compression deformation with damage and decay rates. (**A**) Compression deformation degree–damage rate; (**B**) Compression deformation degree–decay rate.

All images under each compression deformation were processed and calculated, and the average value was taken as the average damage rate under this deformation. The damage rate and storage decay rate of mandarins are shown in Table 1. Regression analysis was used to establish regression equations with compression deformation as the independent variable and damage rate (Y1) and storage decay rate (Y2) as the dependent variables, as shown in Formulas (2) and (3) and Figure 6.

The coefficient of determination R2 of the regression model of compression deformation with damage rate and decay rate was 0.94 and 0.97, respectively, and the coefficient of significance was 0.005 and 0.015, respectively, which showed that the model had good reliability.

Y1 = 0.964X − 0.468(2)

Y2 = 2.625X + 2.5(3)

## 4. Conclusions

This study explored the internal and external mechanical damage process of mandarin under compression and its impact on storage quality. The results showed that when the compression deformation was greater than 8 mm, the mandarin pulp gradually began to rupture. When the compression deformation was greater than 12 mm, the oil glands and guard cells on the surface of mandarin peel were seriously damaged. Additionally, the cavity in the middle of the pulp increased significantly, there was obvious blockage, and the juice sac was damaged, thus inviting bacterial invasion and accelerating the decay of mandarin.

The destruction of the peel and pulp structure led to an increase in cellular respiration intensity and the activity of enzymes such as pectin methylase, in turn accelerating the degradation of polysaccharides, finally resulting in a decrease in the firmness of mandarin. Furthermore, mechanical damage also caused some physiological activities of mandarin to be disordered, resulting in a significant decrease in TSS and TA content. As a result, the amount of decay in the two mandarin treatment groups whose compression deformation was more than 12 mm was much larger than that in other groups during the storage period.

The damage characteristics of mandarin pulp images were extracted, and the damage rate was calculated by image segmentation methods such as threshold segmentation, mask segmentation, and region growth. A linear regression model of mandarin compression deformation with damage rate and storage deterioration rate was established, and the coefficients of determination R^2^ were 0.94 and 0.97, respectively.

Therefore, in actual production, the compression deformation should be lower than 8 mm. Our findings provide the theoretical basis for predicting and reducing decay rate and formulating storage protection strategies of mandarin.

## Figures and Tables

**Table 1:** Damage rate and decay rate of mandarins.

Compression Deformation Degree (mm)	Damage Rate (%)	Decay Rate (%)
4	3.82 ± 0.35 ^a^	15
8	5.74 ± 0.46 ^b^	20
12	10.81 ± 0.89 ^c^	35
16	16.58 ± 1.07 ^d^	45

Note: Same lowercase letters indicate non-significant differences from each other, and different lowercase letters indicate significant differences.

## Data Availability

The original contributions presented in the study are included in the article, further inquiries can be directed to the corresponding author.
